# Improving Photosensitivity and Transparency in Organic Phototransistor with Blending Insulating Polymers

**DOI:** 10.3390/mi14030620

**Published:** 2023-03-08

**Authors:** Hyunji Shin, Dongwook Kim, Jaehoon Park, Dae Yu Kim

**Affiliations:** 1 Department of ICT-Future Vehicle Convergence Education & Research Center, Inha University, Incheon 22212, Republic of Korea; 2 Department of Electrical and Computer Engineering, Inha University, Incheon 22212, Republic of Korea; 3 Center for Sensor Systems, Inha University, Incheon 22212, Republic of Korea; 4 Department of Electronic Engineering, Hallym University, Chuncheon 24252, Republic of Korea; 5 Inha Research Institute for Aerospace Medicine, Inha University, Incheon 22212, Republic of Korea

**Keywords:** organic semiconductors, organic phototransistors, OTFTs, transparent phototransistors, TIPS-Pn

## Abstract

Organic phototransistors exhibit great promise for use in a wide range of technological applications due to their flexibility, low cost, and low-temperature processability. However, their low transparency due to visible light absorption has hindered their adoption in next-generation transparent electronics. For this reason, the present study sought to develop a highly sensitive organic phototransistor with greater transparency and significantly higher light sensitivity in the visible and UVA regions without deterioration in its electrical properties. An organic blended thin-film transistor (TFT) fabricated from the blend of an organic semiconductor and an insulating polymer demonstrated improved electrical properties in the dark and a higher current under light irradiation even though its transmittance was higher. The device exhibited a transmittance of 87.28% and a photosensitivity of 7049.96 in the visible light region that were 4.37% and 980 times higher than those of the single-semiconductor-based device. The carrier mobility of the device blended with the insulating polymer was improved and greatly amplified under light irradiation. It is believed that the insulating polymer facilitated the crystallization of the organic semiconductor, thus promoting the flow of photogenerated excitons and improving the photocurrent. Overall, the proposed TFT offers excellent low-temperature processability and has the potential to be employed in a range of transparent electronic applications.

## 1. Introduction

Recently, transparent electronics have received significant research attention for use in a range of technological innovations, including windscreen technology, smart windows, digital signage, and smart glasses [1,2,3,4]. Transparent electronic devices are capable of providing more data through transparent display systems and acquiring more information through compact sensing systems. Visible light communication (VLC) systems in vehicles, airplanes, and medical facilities have also attracted recent attention because they do not suffer from X-ray, ultraviolet (UV), or infrared (IR) interference [5,6,7,8]. However, systems that detect light in the visible range are not transparent to the human eye, thus transparency and photodetector performance are inevitably in conflict. Detection systems that employ UV and IR can avoid this problem, but they cannot be employed in VLC systems that utilize light-emitting diodes (LEDs) as a light source and are not suitable for organic electronics fabricated via low-temperature, large-area processing for the detection of visible light [9,10,11,12]. Research has thus been conducted on devices that combine fine-grained inorganic materials such as nanoparticles or quantum dots with highly transparent oxide semiconductors [6,13,14,15,16], while the development of extremely thin semiconductor layers could also facilitate the fabrication of highly transparent visible-light phototransistors. However, excessively thin active layers tend to exhibit a poor drain current (I_D_) due to low carrier mobility (μ) and surface non-uniformity [10,17,18,19,20].

In response to these limitations, there has recently been a greater focus on the development of organic electronics. Because small organic molecules and polymers can be easily processed in solution, organic electronics have the potential to be fabricated using low-cost, large-area, low-temperature-processing technology [21,22]. The properties of these materials can also improve the performance of electronic devices via rational material synthesis and molecular design, which can include the doping and/or mixing of materials [23,24,25].

In the present study, by blending 6,13-bis(triisopropylsilylethynyl)pentacene (TIPS-Pn) with polystyrene (PS), we fabricated a highly transparent organic phototransistor with significantly higher light sensitivity in the visible and UVA regions. TIPS-Pn is an organic semiconductor with high stability in the air and high solubility in organic solvents that exhibits good performance even at a low supply voltage [26,27,28]. In addition, the crystal growth of Pn molecules is highly dependent on the surface energy of the substrate; thus, the electrical performance of a device using TIPS-Pn can be readily adjusted by controlling Pn crystallization via the surface energy of the substrate or the evaporation rate of the solvent [29,30,31]. Similarly, PS was employed in the present study as an insulating polymer because of its advantageous binding properties, high insulating ability, and very high transparency [26,32,33]. The fabricated phototransistor exhibited an excellent switching effect and carrier mobility in the dark even though the absolute content of the conductive semiconductor in the active layer was lower due to it being blended with PS. It also demonstrated a maximum transparency of 87.28%, which represents a 4.37% improvement for visible light compared to a conventional single-semiconductor-based device, and the photosensitivity was improved 980-fold under visible light at 450 nm and 2080-fold under UVA at 340 nm. The incorporation of PS increased the grain width of the TIPS-Pn crystals, allowing the rapid propagation of holes and improving the electrical properties and photocurrent of the device. Our highly transparent organic phototransistor with high photosensitivity under visible light and UVA thus exhibits potential for use in VLC systems with LED light sources and compact transparent sensing systems.

## 2. Materials and Methods

**Figure 1:**
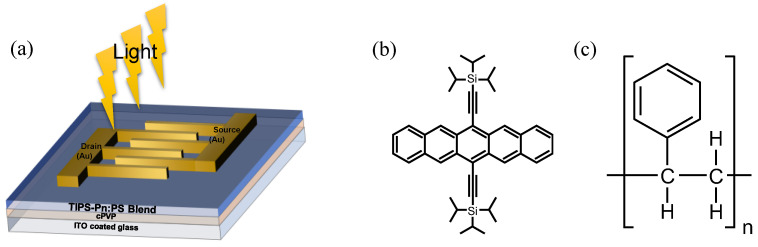
(**a**) Schematic illustration of the fabricated TFT under light irradiation and the chemical structure of (**b**) TIPS-Pn and (**c**) PS.

Figure 1a presents a three-dimensional diagram of a fabricated blended TIPS-Pn:PS thin-film transistor (TFT) under light irradiation, while the chemical structures of TIPS-Pn and PS are displayed in Figure 1b,c, respectively. Indium tin oxide (ITO)-coated glass was used as both the gate electrode and substrate for the bottom-gate/top-contact-structured TFT. Poly(4-vinylphenol) (PVP; Sigma-Aldrich, molecular weight [MW] of 11,000) was dissolved in propylene glycol methyl ether acetate together with poly(melamine-co-formaldehyde) methylated as a cross-linking agent at equal proportions (5 wt%). This solution was spin-coated onto the ITO layer at 2000 rpm for 30 s and then annealed at 210 °C for 30 min as a cross-linked PVP (cPVP) insulating layer. A 1 wt% TIPS-Pn solution was then blended with 1 wt% PS (Sigma-Aldrich) with two different MW (90,000 or 280,000) that had been dissolved in anisole at a TIPS-Pn:PS weight ratio of 1.5:1 and 2:1. The resulting blends (denoted as Blend 90k 1.5:1, Blend 280k 1.5:1, Blend 90k 2:1, and Blend 280k 2:1) were spin-coated onto the cPVP layer at 1000 rpm for 35 s before being dried in ambient air for 1 h. Following this, a 50 nm thick Au source and drain electrodes were thermally deposited using a finger-typed shadow mask with a channel width (W) and length (L) of 2000 and 80 μm, respectively.

The TFTs were irradiated with monochromatic light generated using a xenon lamp (450 W) through an optical fiber to measure their electrical characteristics for specific wavelengths of light. The wavelengths of the light were set at 690, 450, and 340 nm using a MonoRa-320i monochromator (Dongwoo Optron Co., Ltd., Gwangju-si, Korea). To ensure that the generation of excitons in response to the irradiation was uniform for each TFT, all of the TFTs were exposed to light for 1 min before measurements were taken. The recovery current, which was used to assess the extent to which the higher current due to light absorption returned to the initial dark current when the TFTs no longer received light, was measured by blocking the light for 4 min. The electrical properties of the TFTs obtained under dark and irradiated conditions were measured using an ELECS421C semiconductor analyzer.

## 3. Results

### 3.1. Dark Conditions

TIPS-Pn and two types of PS (i.e., with an MW of 90,000 or 280,000) were blended at ratios of 1.5:1 (Blend 90k 1.5:1 and Blend 280k 1.5:1) and 2:1 (Blend 90k 2:1 and Blend 280k 2:1) to produce TFTs, which were then compared with a pristine TIPS-Pn TFT.

**Figure 2:**
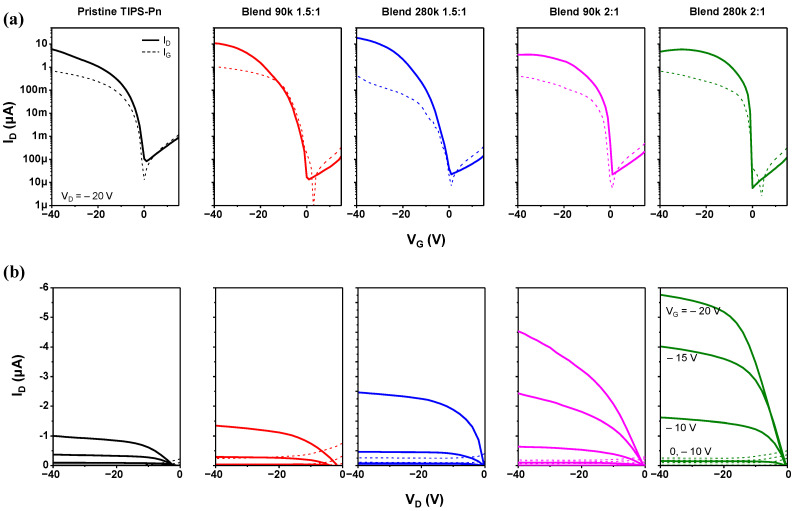
(**a**) Transfer and (**b**) output characteristics of the fabricated TFTs under dark conditions.

Figure 2a shows the transfer characteristics of the pristine TIPS-Pn, Blend 90k 1.5:1, Blend 280k 1.5:1, Blend 90k 2:1, and Blend 280k 2:1 TFTs measured in a dark box. In our experiment, the drain voltage (V_D_) was fixed at −20 V, and the gate voltage (V_G_) was varied from 15 to −40 V in steps of 1 V. All of the fabricated TFTs turned on at a V_G_ of around 0, and the current on/off ratio (I_on/off_) was over 10^4^.

Table 1 presents the parameters extracted from the transfer characteristics, showing that the I_on/off_ ratio of the blended TFTs was over 10^5^_,_ significantly higher than that of the pristine TIPS-Pn TFT. The TFTs blended at a ratio of 1.5:1 had significantly higher carrier mobilities of 0.07 and 0.12 cm^2^/V∙s compared with the pristine TIPS-Pn TFT, while those of the Blend 90k 2:1 and Blend 280k 2:1 TFTs were the same or only slightly higher (0.03 and 0.05 cm^2^/V∙s, respectively). The threshold voltage (V_T_) of the TFTs blended at 1.5:1 was similar to that of the pristine TFT, but those of the Blend 90k 2:1 and Blend 280k 2:1 TFTs were 5.58 and 9.30 V higher, respectively. The output characteristics of the TFTs under dark conditions are presented in Figure 2b. At V_G_ values of 0, −5, −10, −15, and −20 V, V_D_ was swept from 0 to −40 V in increments of −1 V. The saturation of I_D_ was observed for all of the fabricated TFTs, with the I_D_s of the Blend 90k 2:1 and Blend 280k 2:1 TFTs significantly higher than that of the pristine TFT.

**Figure 3:**
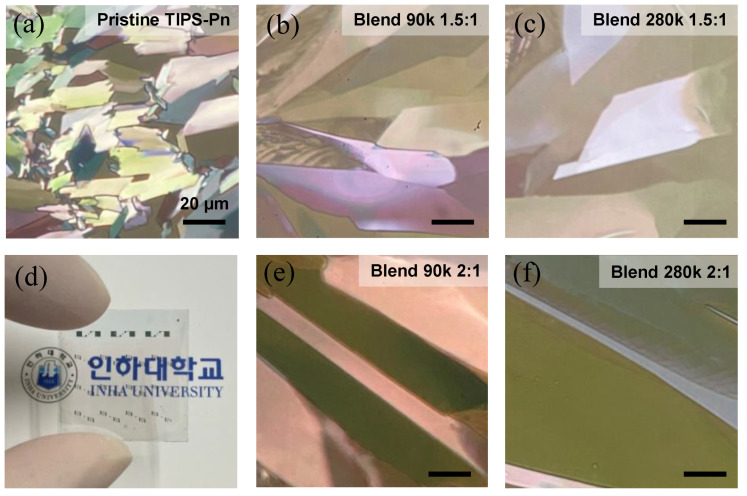
Polarized optical microscope images of spin-coated (**a**) pristine TIPS-Pn, (**b**,**e**) TIPS-Pn/PS (90,000 MW) blend, and (**c**,**f**) TIPS-Pn/PS (280,000 MW) blended films with blend ratios of (**b**,**c**) 1.5:1 and (**e**,**f**) 2:1, and (**d**) an optical image of the Blend 280 k 2:1 TFTs.

Polarized optical microscope (POM) images taken from above the channel region of the fabricated TFTs are displayed in Figure 3. Figure 3a shows that the surface of the active layer for the pristine TFT had a random distribution of small TIPS-Pn crystals. The grain size of these crystals became larger when blended with 90 kg/mol PS and larger still when blended with 280 kg/mol PS (Figure 3b–f). However, with the 2:1 TIPS-Pn:PS blends, the 1.5:1 blends exhibited a lighter grain color with less pronounced borders. The molecular alignment and crystallinity of TIPS-Pn can be improved by lowering the solvent evaporation rate, resulting in an improved carrier mobility and I_on/off_ ratio [34,35,36,37]. Compared with the pristine TIPS-Pn film, which had the smallest grain width, the increased viscosity of the solution and the improved molecular arrangement due to the addition of PS increased the grain width of the crystals in the blended films, thus increasing the carrier mobility. This improvement was greater for the 1.5:1 blends than for the 2:1 blends, and greater for the 280k blends than for the 90k blends. According to Zajaczkowska et al. [37], the higher the MW of the blended PS, the larger the domain of the crystals that are generated.

The V_T_ of the TFTs blended at a 2:1 ratio was more positive than that of the pristine TFT, which indicates that greater charge trapping occurred at the semiconductor–insulator interface [38]. According to Jang et al. [29], cPVP, which was used as the insulating layer in our devices, retains many hydroxyl groups even after the crosslinking process, but PS can reduce the number of trapping sites by blocking the hydroxyl groups remaining on the surface of the cPVP. Therefore, the 1.5:1 TFTs likely blocked the hydroxyl groups on the surface of the cPVP insulation layer more effectively than did the 2:1 TFTs [29,39]. In addition, the 2:1 TFTs, which had a higher absolute TIPS-Pn content than the 1.5:1 TFTs, exhibited a higher I_D_, even though the V_T_ was more positive. This is because the charge carriers in the well-crystallized blend films were likely to have propagated more quickly in the larger crystals and the number captured and released at the grain boundaries would have been relatively low. Figure 3d presents an optical image of 24 Blend 280k 2:1 TFTs fabricated on a 2 cm × 2 cm glass substrate with a very high transparency.

### 3.2. Under Light Irradiation

**Figure 4:**
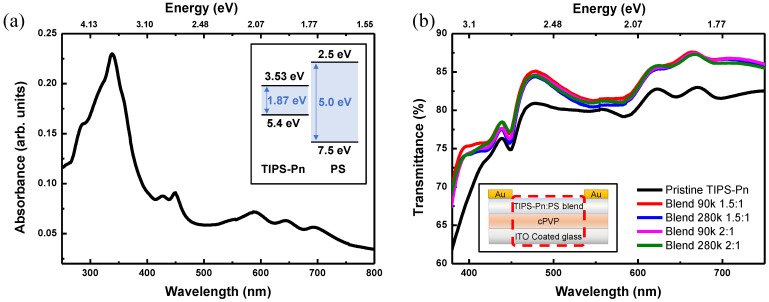
(**a**) UV/visible spectrometer measurements for the TIPS-Pn thin film (inset: energy band diagrams of TIPS-Pn and PS) and (**b**) transmittance in the visible light range (380–750 nm) for the pristine TIPS-Pn and four types of blended TIPS-Pn/PS TFTs in the channel region excluding the source/drain electrode (inset: schematic diagram showing the area where the transmittance was measured, indicated by the red dotted line).

The absorption spectrum of the pristine TIPS-Pn thin film spin-coated on a quartz substrate was measured for a wavelength range of 250–800 nm (Figure 4a).

A low absorbance peak for the TIPS-Pn thin film appeared at 690 nm, followed by a distinct thin peak at 450 nm and a maximum at 340 nm. While TIPS-Pn has an energy band gap of 1.87 eV, PS has a high energy band gap of 5.0 eV, corresponding to a wavelength of 248 nm, meaning that electron excitation cannot occur within PS film under visible or UVA light [40,41]. Therefore, the wavelengths of 690, 450, and 340 nm were selected for light irradiation to measure the electrical characteristics of the fabricated blended TFTs and examine the absorption–current correlation and the recovery current after light irradiation had been removed. Figure 4b presents the transmittance in the visible light region (380–750 nm) of the channel region for the fabricated TFTs. The transmittance of the pristine TIPS-Pn TFT under visible light ranged from a minimum of 61.97% to a maximum of 82.99%, whereas the transmittance of the blended TFTs was higher than that of the pristine TFT. In particular, the transmittance of the Blend 90k 1.5:1 TFT exhibited a minimum of 70.1% and a maximum of 87.61%.

**Figure 5:**
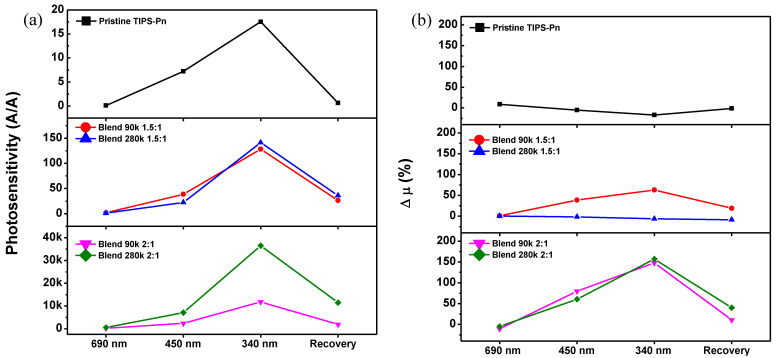
(**a**) Photosensitivity and (**b**) field-effect mobility of the fabricated TFTs under irradiation light of 690, 450, and 340 nm and recovery to the initial dark state.

Figure 5 shows the photosensitivity and the rate of change in the carrier mobility (Δ*μ*) of the pristine and blended TIPS-Pn TFTs under different wavelengths of incident light and under dark conditions. These parameters were calculated using the following equations:

(1) $$P=\frac{I_{Ph}}{I_{Dark}}=\frac{I_{Light}-I_{Dark}}{I_{Dark}}$$

(2) $$\mu =\frac{2L}{WC_{i}}{\left(\frac{d\sqrt{I_{D}}}{dV_{G}}\right)}^{2}$$

(3) $$\Delta \mu \;\left(\%\right)=\frac{\mu_{Light}-\mu_{Dark}}{\mu_{Dark}}\times 100$$

where *P* is the photosensitivity, *I_Ph_* is the light-induced *I_D_*, *I_Light_* and *I_Dark_* are the I_D_ under the light and dark conditions, respectively, and *C_i_* is the capacitance per unit area of the basic insulator [42,43,44]. The photosensitivity was determined to be the highest value according to *V_G_*, and it has been found useful in the past to evaluate the current amplification for TFTs under light irradiation.

When irradiated with 690 nm light, negligible photosensitivity was observed for all of the TFTs (Figure 5a). As shown in Figure 4a, irradiation with monochromatic light of 690 nm, which is longer than the energy band gap of TIPS-Pn, may have been insufficient for TIPS-Pn to generate excitons. In contrast, the TIPS-Pn TFT exhibited a photosensitivity of 7.21 under light irradiation of 450 nm, and all of the blended TFTs also exhibited a significant increase in photosensitivity compared to under 690 nm light, with the Blend 280k 2:1 TFT showing the greatest increase (7049.96). The photosensitivity improved further under light of 340 nm, which is the wavelength of maximum absorbance for TIPS-Pn; in particular, a photosensitivity of 36,480.68 was observed for the Blend 280k 2:1 TFT. Because the absolute amount of TIPS-Pn was lower for the 1.5:1 TFTs than for the pristine TIPS-Pn or 2:1 TFTs, the probability that fewer exciton pairs were generated is very high. The significantly larger photosensitivity exhibited by the blended TFTs compared with the pristine TFT suggests that blending with PS contributed more to the photocurrent flow than to the quantitative improvement in the photocurrent. In other words, the increase in photocurrent was likely due to improvements in the charge transport pathways than to the generation of more exciton pairs. In particular, when exciton pairs are generated by light irradiation in the TIPS-Pn thin film, electrons fill the positively charged states and holes propagate rapidly along the crystals to join the I_D_ and increase the photocurrent.

The blended TFTs with well-crystallized TIPS-Pn exhibited a significant increase in both the dark current and photocurrent, though the 2:1 blends outperformed the 1.5:1 blends. In the dark, the photosensitivity of the TFTs recovered to a level close to their initial dark state, but the Blend 90k and 280k 2:1 TFTs, which has a greatly improved photosensitivity, exhibited a slower recovery. The recovery photosensitivities of the two 2:1 blends were higher than those of the pristine TIPS-Pn and the 1.5:1 blends, with the Blend 280k 2:1 TFT having a significantly higher recovery photosensitivity of 11,506.85. These observations are in accordance with the behavior of V_T_ under dark conditions, and the incomplete recovery of photosensitivity is closely related to charge traps, as with V_T_. In other words, it is likely that the exciton pairs generated under light irradiation were trapped in deep states and could not easily escape; thus, they failed to recombine within a sufficient time period, resulting in incomplete recovery [39].

The Δ*μ* was also negligible for all of the fabricated TFTs under light irradiation of 690 nm (Figure 5b). The Blend 90k 1.5:1 TFT exhibited greater charge mobility under 450 and 340 nm light irradiation (39% and 63%, respectively), while pristine TFT had a negligible change in mobility under both light irradiation and during recovery. The Blend 280k 1.5:1 TFT also exhibited no significant change in mobility even under light irradiation, which was due to the high mobility of the device observed under dark conditions. However, the charge mobility of both 2:1 TFTs under 450 and 340 nm light greatly improved. In particular, the Δ*μ* of the Blend 90k 2:1 TFT increased by 80% at 450 nm and 148% at 340 nm, while that of the Blend 280k 2:1 TFT increased by 60% and 157%, respectively.

**Figure 6:**
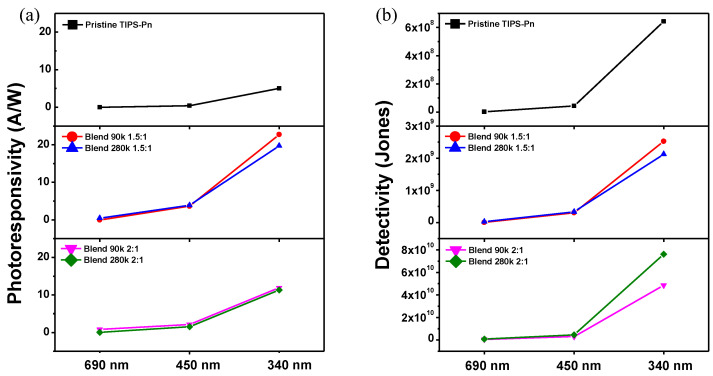
(**a**) Photoresponsivity and (**b**) detectivity of the fabricated TFTs under irradiation light of 690, 450, and 340 nm.

Equations (4)–(6) describe the photoresponsivity, detectivity, and external quantum efficiency (EQE) of the phototransistors, respectively:

(4) $$R=\frac{J_{Ph}}{P_{in}}=\frac{I_{Ph}}{P_{in}A}$$

(5) $$D^{*}=\frac{R}{\sqrt{2qJ_{Dark}}}=R\sqrt{\frac{A}{2qI_{Dark}}}$$

(6) $$EQE=\frac{h\nu }{q}R=\frac{hc}{q\lambda }R$$

where *R* is the photoresponsivity, *J_Ph_* is the photocurrent density (*I_ph_* per effective area), *P_in_* is the incident light power density, *A* is the channel area, D* is the detectivity, *q* is the unit charge, *J_Dark_* is the dark current density, *h* is Planck’s constant, *ν* is the incident light frequency, *c* is the light speed, and *λ* is the irradiated wavelength [45,46,47]. Along with photosensitivity, these parameters are widely used indicators for the photocurrent response of phototransistors. The results for these indicators for the fabricated TFTs are summarized in Figure 6 and Table 2.

As shown in Figure 6a, the photoresponsivity was low at 690 nm, which is longer than the wavelength for the energy band gap of TIPS-Pn. However, the photoresponsivity of all of the blended TFTs was higher than that of the pristine TFT under incident light of 450 and 340 nm. At 340 nm, the pristine TIPS-Pn TFT had a photoresponsivity of 4.99 A/W, compared with 22.71 A/W for the Blend 90k 1.5:1 TFT and 19.73 A/W for the Blend 280k 1.5:1 TFT, a 4.55- and 3.95-fold improvement, respectively. However, unlike the photosensitivity, the two devices with a blend ratio of 2:1 had lower photoresponsivity than the TFTs with a blend ratio of 1.5:1. This is because the photoresponsivity depends on the intensity of the incident light regardless of I_D_ under dark conditions, while photosensitivity is related to the dark state I_D_ (Equations (1) and (4)). On the other hand, the detectivity reflects both *I_Dark_* and the incident light power intensity (Equation (5)). Of the TFTs irradiated with 450 and 340 nm light, it was observed that the two blended TFTs with a ratio of 1.5:1 had a higher detectivity than the pristine TFT. In addition, the two TFTs with a 2:1 blend ratio exhibited an even higher detectivity (Figure 6b). In particular, the detectivity under 340 nm light was 4.86 × 10^10^ Jones for the Blend 90k 2:1 TFT and 7.63 × 10^10^ Jones for the Blend 280k 2:1 TFT, which was the highest of the fabricated TFTs.

Table 2 summarizes the EQE for the fabricated TFTs calculated using Equation (6) for the three tested wavelengths of light. All of the fabricated TFTs exhibited only a minor response to 690 nm light. The pristine TFT had an EQE of 108% following irradiation with 450 nm light, which was lower than that of the blended TFTs. In particular, the Blend 280k 1.5:1 TFT had the highest EQE of 1072% under 450-nm light. The pristine TFT had an EQE of 1821% under 340 nm light, but this was still considerably lower than that of the blended TFTs. The Blend 90k 1.5:1 TFT exhibited a particularly high EQE of 8284% under 340 nm light.

## 4. Conclusions

In this study, the correlation between the light absorption and electrical properties of TIPS-Pn-based TFTs blended with PS was investigated for the purpose of developing highly transparent organic phototransistors with excellent photosensitivity. The TIPS-Pn:PS TFTs fabricated under various blending conditions exhibited a higher I_on/off_ ratio and output current compared to the pristine TIPS-Pn TFT despite having a lower semiconductor content. Under light irradiation, the blended TFTs exhibited a significant improvement in photosensitivity compared to the pristine TIPS-Pn TFT. In particular, the Blend 280k 2:1 TFT had a photosensitivity that was 980 times higher under 450 nm visible light irradiation and 2080 times higher under 340 nm UVA irradiation compared with the pristine TFT. At the same time, the transmittance of this TFT was remarkably high, with a minimum of 69.52% (at 380 nm) and a maximum of 87.28% (at 667 nm), which were 7.55% and 4.37% higher than those of the TIPS-Pn TFT, respectively. This improvement in transparency indicates that the TIPS-Pn content was reduced by blending it with PS, and the grain width of the crystals increased during the TIPS-Pn crystallization process within the thin film. Accordingly, the blended TIPS-Pn:PS TFTs exhibited both improved transmittance and superior photocurrent, thus overcoming the conventional trade-off between the two observed for organic phototransistors.

The proposed blended TFTs respond to specific spectra of visible light and UVA and are amenable to low-temperature solution processing. However, these TFTs exhibited incomplete recovery of photosensitivity compared to the pristine TFT, probably due to deep-state trapping. These devices thus require the optimization of the shift in V_T_ and the decay in photosensitivity over time in relation to the trap density that arises due to blending with PS. If this research is conducted, our device has the potential to be applied to next-generation optoelectronic systems that require highly transparent light sensing such as vehicle infotainment, window-integrated displays, HMI displays, and artificial optic nerve systems.

## Figures and Tables

**Table 1:** Electrical properties of the TFTs fabricated with pristine TIPs-Pn, Blend 90k 1.5:1, Blend 280k 1.5:1, Blend 90k 2:1, and Blend 280k 2:1 extracted from the transfer characteristics under dark conditions.

Parameters	Pristine TIPS-Pn	Blend 90k 1.5:1	Blend 280k 1.5:1	Blend 90k 2:1	Blend 280k 2:1
I_on/off_ (A/A)	7.17 × 10^4^	7.94 × 10^5^	8.18 × 10^5^	2.44 × 10^5^	1.02 × 10^6^
μ (cm^2^/V∙s)	0.03	0.07	0.12	0.03	0.05
V_T_ (V)	−9.77	−10.85	−11.58	−4.19	−0.47
SS (V/decade)	1.41	0.88	1.94	1.08	0.33

**Table 2:** EQE for the fabricated TFTs under irradiation light of 690, 450, and 340 nm calculated using Equation (6).

	EQE (%)				
Wavelength (nm)	Pristine TIPS-Pn	Blend 90k 1.5:1	Blend 280k 1.5:1	Blend 90k 2:1	Blend 280k 2:1
690	0.004	0.114	83	145	6
450	108	997	1072	588	432
340	1821	8284	7197	4342	4119

## Data Availability

The data presented in this study are available on request from the corresponding author.
